# The miscarriage circle of care: towards leveraging online spaces for social support

**DOI:** 10.1186/s12905-022-01597-1

**Published:** 2022-01-29

**Authors:** Mona Y. Alqassim, K. Cassie Kresnye, Katie A. Siek, John Lee, Maria K. Wolters

**Affiliations:** 1 School of Informatics, University of Edinburgh, 10 Crichton St, Edinburgh, UK; 2 School of Informatics, Computing, and Engineering, Indiana University, Bloomington, IN USA; 3 The Alan Turing Institute for Data Science and Artificial Intelligence, London, UK; 4 Institute of Design Informatics, School of Informatics, University of Edinburgh, 10 Crichton St, Edinburgh, UK

**Keywords:** Miscarriage, Pregnancy loss, Social support, Care network, Circle of care, eHealth, mHealth

## Abstract

**Background:**

Lack of social support during and after miscarriage can greatly affect mental wellbeing. With miscarriages being a common experience, there remains a discrepancy in the social support received after a pregnancy is lost.

**Method:**

42 people who had experienced at least one miscarriage took part in an Asynchronous Remote Community (ARC) study. The study involved 16 activities (discussions, creative tasks, and surveys) in two closed, secret Facebook groups over eight weeks. Descriptive statistics were used to analyse quantitative data, and content analysis was used for qualitative data.

**Results:**

There were two main miscarriage care networks, formal (health care providers) and informal (friends, family, work colleagues). The formal care network was the most trusted informational support source, while the informal care network was the main source of tangible support. However, often, participants’ care networks were unable to provide sufficient informational, emotional, esteem, and network support. Peers who also had experienced miscarriage played a crucial role in addressing these gaps in social support. Technology use varied greatly, with smartphone use as the only common denominator. While there was a range of online support sources, participants tended to focus on only a few, and there was no single common preferred source.

**Discussion:**

We propose a Miscarriage Circle of Care Model (MCCM), with peer advisors playing a central role in improving communication channels and social support provision. We show how the MCCM can be used to identify gaps in service provision and opportunities where technology can be leveraged to fill those gaps.

## Background

### Objective

Miscarriage is a common type of pregnancy loss that affects one in five pregnancies (15–20%) [1–8]. One in a hundred couples experience consecutive miscarriages (recurrent miscarriage, [9, 10]). While trans/masculine and non-binary people also experience miscarriage [11, 12], the remainder of this paper will focus on the experience of cisgender women, given that this was how the participants in our study identified.

Previous studies identified the importance of social support for the well-being of those who have miscarried (e.g., [13]), and described the experience of social support after miscarriage (e.g., [14]). In this study, we investigate the social support needs for those who have miscarried from a holistic perspective that integrates formal and informal care networks, and the role of online spaces in social support provision. The results support formal modelling for health service design that can connect women to appropriate sources of social support seamlessly, while preserving confidentiality.

Specifically, our research questions are:

1. Who are the key people in the care network of women who have miscarried?
2. How do women use social support and communicate with their care network to cope with miscarriage?
3. How can eHealth solutions be leveraged to improve communication about social support?

### Miscarriage and women’s wellbeing

Although miscarriage is an unpredictable event, those who have miscarried often view it as a personal failure. They may blame and punish themselves and cite their lifestyle and habits as a cause [15–18]. Up to 5% of women who have miscarried experience post-traumatic stress symptoms [19]. The loss can trigger anxiety and depression [20], anger, and the urge to self-harm [19]. Women may grieve deeply for years after miscarriage [21, 22], and the grief reaction can be as intense as that due to perinatal death [8, 15, 16, 23–25].

Appropriate social support for women who have experienced a miscarriage can provide a much needed boost to their wellbeing both in the short term and in the long term [19, 26]. The more satisfied women are with the level of support received, the less likely they are to experience mental health issues [13, 22, 27, 28]. Unfortunately, many women are unhappy with the support they receive from their care network after their loss [13, 29].

While previous studies identified the importance of social support for the physical and psychological wellbeing of women who have miscarried, little is known about the dimensions, amount, and categories of appropriate social support needs of women who have miscarried. In particular, there is a need to identify which social support is preferred or desired, and by which members of women’s care networks it should be provided.

### Dimensions of social support

Social support has been defined as “verbal and nonverbal communication between recipients and providers that reduces uncertainty about the situation, the self, the other, or the relationships, and functions to enhance a perception of personal control in one’s experience” [30, p. 19]. Social support is most effective when it matches the needs and preferences of the people who require it, and is perceived by them to be helpful [31, 32]. For the purpose of this study, we adopt a definition of social support [32–38] that has proved useful in previous studies of online social support for people living with rare diseases [39] or stigmatised conditions, such as being HIV positive [40].

Following [33], there are two main types of social support, action-facilitating and nurturant. Action-facilitating support is intended to eliminate or actively solve the problems faced by the person who needs support which includes informational and tangible types of support. Nurturant support is intended to provide consolation and comfort without actively solving the problem or facilitating action which includes emotional, esteem, and network types of support. Table 1 presents an explanation of each of those sub-types of social support [33, 34, 41–44].

**Table 1:** Types of Social Support

Support type	Examples
Informational	*Suggestions/advice*: to suggest actions and provide guidance
	*Referral*: referring to another source of information
	*Teaching*: to explain facts or teach skills needed
Emotional	*Empathy*: to feel sorrow or empathy
	*Understanding*: to understand the situation of the participants
Esteem	*Validation*: to express agreement with participants perspective on the situation
	*Compliment*: positive comments about participants
Network	*Access*: to provide access to peers
	*Friendship*: request to make connections or offer to talk
	*Peer*: to be reminded of the availability of existing peers
Tangible	*Gift*: to give participants something
	*Direct tasks*: to perform a task directly for participants

### Care networks as a source of social support

Social support may be provided through ***Formal*** and ***Informal*** care networks. Formal networks include professional service providers, such as doctors, obstetricians, midwives, and therapists, who are professionally trained and paid for their work. The informal care network involves those who are in a social relationship with the person who receives care and who are not paid for their work, such as family, friends and communities [45–49].

In practice, social support for those who have miscarried is often inconsistent or insufficient [8]. Communication between those who have miscarried and their care network, including health care providers, is often difficult [50–53]. A major barrier to receiving appropriate social support is that care network members often do not understand the emotional complexities involved, and prioritise physical wellbeing over psychological needs [19, 53]. While those who have experienced miscarriage seek emotional support by talking to their care network after miscarriage, they often feel unable to express the negative emotions related to their loss, and wish their care network would be more sensitive [20, 54, 55]. This is partially due to the silence surrounding the topic of miscarriage [56]. As a result, miscarriage continues to be stigmatised and misunderstood [57].

There are many sources of specialised social support for miscarriage, such as counselling, supportive listening [58], and in-person support groups [59]. However, a single intervention is typically not enough. There may also be long waiting lists for interventions, such as counselling. In-person services can be hard to access if the person who has experienced miscarriage has responsibilities that prevent access [60], such as caring for a family member or rigid work schedules.

### eHealth for miscarriage support

eHealth is a promising alternative for those who find accessing in-person services difficult [61]. Kersting et al. [62] evaluated an internet-based psychotherapy programme with people coping with a pregnancy loss. The system facilitated anonymous and asynchronous communication across a wide geographic area. The level of grief for women who miscarried decreased immediately after the treatment and depression symptoms decreased during the 3-month follow-up session [62]. Recent relevant systematic reviews [63, 64] show that Kersting et al.’s programme is still the only eHealth psychosocial support intervention for women who have miscarried that has been assessed in a randomised controlled trial.

Women who experience miscarriage also use online discussion forums to seek support and information [29, 65, 66]. On such online forums and other social media platforms, disclosure of information is usually managed carefully. Andalibi et al. describe the phenomenon of “network-level reciprocal disclosure” (NLRD), where once an individual observes others’ disclosure, they are motivated to disclose in a safe space within their network [57]. Building on this work, Andalibi and Forte [67] designed a prototype mobile application which embodies the NLRD model using pregnancy loss as a case study. However, it is not clear how solutions such as NotAlone, which emphasise the informal care network, would integrate with more standard models of care [64], which emphasise the formal care network and focus on counselling.

### Shifting towards a circle of care model

The Circle of Care Model (CCM) provides a coherent framework for integrating formal and informal care networks. CCM was first developed as a user requirements elicitation method to improve palliative care for patients at the end of their lives [68]. The person who receives support and care is firmly placed at the centre of their own Circle of Care, and those who give care, be it formal or informal, are then linked to the person in the centre by role [68, 69]. A CCM covers four elements [70], which are related to each other through a patient and their needs:

1. The *person*,
2. The people that are involved in that person’s care (*providers*),
3. The *communication* between network members (person and providers), and
4. The *information repositories* that store information about that person.

CCM has been successfully used to model the coordination of complex networks which involve continuity of care [70, 71]. It is useful for highlighting gaps in communication, both between providers and between providers and the person at the centre, which can lead to adverse events [72]. In this paper, we focus on the providers of social support, and on the communication between the woman who has miscarried and her network of formal and informal providers. Aspects of our work that are relevant to information repositories are discussed in more detail elsewhere [73].

## Methods

In this study, we used the Asynchronous Remote Community (ARC) method to engage with women who have experienced a miscarriage. ARC was designed to connect participants regardless of location or availability to meet in person, and mitigate perceived stigma [74–77]. The method has been used successfully with pregnant women and new mothers [75].

In an ARC study, participants take part in a series of moderated activities over several weeks in private Facebook groups that are set to secret so that they cannot be found by others, and private so that they can only be accessed by invitation.

Activities may include surveys, free text responses, or uploading images. Each research question is typically covered by several activities, which can then be used to triangulate findings [78].

ARC was chosen because it has several advantages:

1. It provides a forum where women who have experienced miscarriage can become familiar with each other over time, thereby facilitating disclosure [57];
2. It allows us to bring together women from different locations;
3. It allows women to participate at any time of their choosing, which lowers barriers to participation; and
4. Participants can choose whether to take part in each activity, and when to engage with an activity.

We recruited participants from two English speaking countries with substantially different health care systems, the United Kingdom, and the United States to increase the generalisability of results across healthcare systems. Preliminary findings regarding social support were reported in [79]. The method itself is further discussed in [77], and findings from activities that target informational support needs are discussed in [73].

### Participants

Participants were recruited from the United Kingdom and the United States. Recruitment was mainly through relevant Facebook groups and personal networks in Facebook. In the UK, we also recruited via leaflets distributed with permission in libraries, charity shops, and local UK Miscarriage Association groups. Participants received compensation in the form of an Amazon gift card (£35 or $50) for their participation in the study, regardless of their level of engagement.

A total of 66 people expressed interest in joining the study and 44 (66%) participants completed the informed consent process. We created two separate, secret Facebook groups, one for participants who had a live birth after their last miscarriage (*Live Birth, LB*) and the other for those who did not (*No Live Birth, No LB*). During the first weeks of the study, two participants from the Live Birth group stopped taking part in activities, one due to family issues, the other without providing a reason. A total of 42 participants completed the ARC study.

Demographic data is summarised in Table 2. Precisely, 57% of participants were from the US and 43% were from the UK. 40 participants identified as women, while one participant preferred not to disclose gender. Participants in the Live Birth group were predominantly from the US ($\chi ^2(1)=30.136, p<0.0001)$, while participants in the No Live Birth group were balanced across locations. Since none of the participants explicitly identified as non-binary or trans/masculine, we will henceforth refer to the study participants as women. Most participants identified as White. In both groups, the majority of participants had a graduate or postgraduate degree, and most were in full or part-time employment. While participants logged into Facebook almost daily, two thirds posted once a week or less.

**Table 2:** Demographics

	NoLB (n = 20)	LB (n = 21)	Total
	N (%)	N (%)	
**No data**	1 (5)	0 (0)	
**Location**			
United Kingdom	9 (45)	8 (38)	17
United States	11 (52)	13 (61)	24
**Age group**			
25-29	2 (10)	3 (14)	5
30-34	6 (30)	5 (24)	11
35-39	6 (30)	9 (42)	15
40-44	3 (10)	3 (14)	6
45+	3 (15)	1 (5)	4
Ethnicity ‘*’			
White	14 (90)	18 (76)	32
Arab	3 (14)	1 (5)	4
Asian	1 (5)	1 (5)	2
Hispanic	1 (5)	0	1
Native American	1 (5)	0	1
Black	0	1 (5)	1
Other	1 (5)	0	1
**Education**			
Postgraduate	6 (30)	6 (19)	12
Graduate	6 (30)	7 (22)	13
other	8 (40)	8 (40)	16
Employment ‘*’			
Full time	10 (45)	10 (45)	20
Part time	4 (35)	3 (14)	7
Other	7 (35)	8 (38)	15
**Self-reported Facebook log in frequency**			
Daily	18 (90)	20 (95)	38
4-6 times per week	1 (5)	1 (5)	2
Once a week	1 (5)	0	1
**Self-reported Facebook posting frequency**			
Daily	1 (5)	3 (14)	4
4-6 times per week	2 (10)	2 (10)	4
2-4 times per week	2 (14)	3 (14)	5
Once a week	5 (24)	2 (10)	7
Rarely	10 (47)	11 (52)	21
**Self-reported Smartphone usage frequency**			
Daily	20 (100)	21 (100)	41

No LB = No Live Birth. LB= Live Birth

‘*’ Indicated fields where multiple options could be selected, resulting in totals greater than participant count

### Activities

The ARC study consisted of 16 activities inspired by [75], and summarised in Table 3. We posted two activities per week over 8 weeks. More details can be found in [79]. The initial and final activities (A1, A2, and A16) focused on study management and group facilitation (e.g., an icebreaker to introduce participants to each other). There were thirteen research activities (A3–A15, c.f. Table 3). These involved writing about participants’ own experiences (Free Text, n = 5), surveys (Survey, n = 6), and posting a self-made drawing that the participant created (Media, n = 2). Free text and media activities were shared in the group, while surveys were private. Each research activity was triangulated [78] with at least one other activity, which served as an internal check of validity of our findings. The six activities we focus on in this paper related directly to social support and technology usage. They are are marked in grey in Table 3. Since women were free to complete activities as they wished, completion rates ranged from 47 to 95%. The No Live Birth (NoLB) group was less active than the Live Birth (LB) group. More detail about the relevant activities is given in “Results” section, as we discuss the findings that pertain to each research question.

**Table 3:** Activities used in study

Week	Activity	Type	N (%)	Live birth N (%)	No live birth N (%)
1	A1: Meet and Greet	FT	37 (88)	19 (90)	18 (85.7)
	A2: Facebook Availability	Poll	38 (90)	18 (85.7)	20 (95)
2	A3: Drawn Timeline	M	36 (86)	19 (90)	17 (81)
	A4: Emotional Wellbeing Scale	S	40 (95)	21 (100)	19 (90)
3	A5: Dear Abby	FT	28 (66)	15 (71)	13 (62)
	A6: Miscarriage Experience	FT	32 (76)	18 (85.7)	14 (66)
4	**A7: Circle Diagram**	M	22 (50)	13 (62)	9 (42.8)
	**A8: Brief COPE**	S	36 (88)	20 (95)	16 (76)
5	**A9: Social Communication**	FT	22 (52)	13 (62)	9 (42.8)
	**A10: Social Support**	S	35 (83)	19 (90)	16 (76)
6	**A11: Coping Mechanisms**	FT	25 (57)	14 (66)	11 (52)
	A12: Missing Information	FT	21 (47)	13 (62)	8 (38)
7	A13: Importance of Information	S	30 (71)	17 (81)	13 (62)
	**A14: Technology Use**	S	33 (79)	18 (85.7)	15 (71)
8	A15: Future Technology	S	34 (81)	19 (90)	15 (71)
	A16: Goodbye	FT	4 (10)	2 (9.5)	2 (9.5)

T= Type of the Activity (FT= Free Text, S= Survey, M= Media). N= Numbers of participants who completed the activity. The activities we focus on in this paper are highlighted in bold

At the end of the study, both groups remained open to avoid harm to participants by ending supportive relationships they may have formed throughout the study. We encouraged participants to review Facebook’s privacy policy for how their information would be used both at the beginning and at the end of the study, when data collection was completed.

### Data analysis

We used R^1^ to analyse participants’ demographic data and for descriptive statistics and non-parametric tests of differences between groups. Content analysis was used to analyse qualitative data. Content analysis is a structured method for coding that focuses on describing who said what to whom, and with what effect [80–82]. Two researchers (MA and CK) independently coded the data using a combination of bottom-up (inductive) content analysis with top-down (deductive) codes derived from the research questions. Based on the initial codes, (MA and CK) agreed on a codebook, and the data were then re-coded. Interrater agreement was $\kappa =0.89$ on the overall coding of each code [83]. Dedoose^2^ was used to coordinate the coding. The resulting codes were then grouped and, where appropriate, mapped to relevant categories from the literature. The five social support types defined by Cutrona and colleages [32, 33], informational, emotional, esteem, network, and tangible support (c.f. Table 1), emerged as the best match for the categories found.

## Results

### RQ1: key people in the care network

#### Data sources

**Fig. 1:**
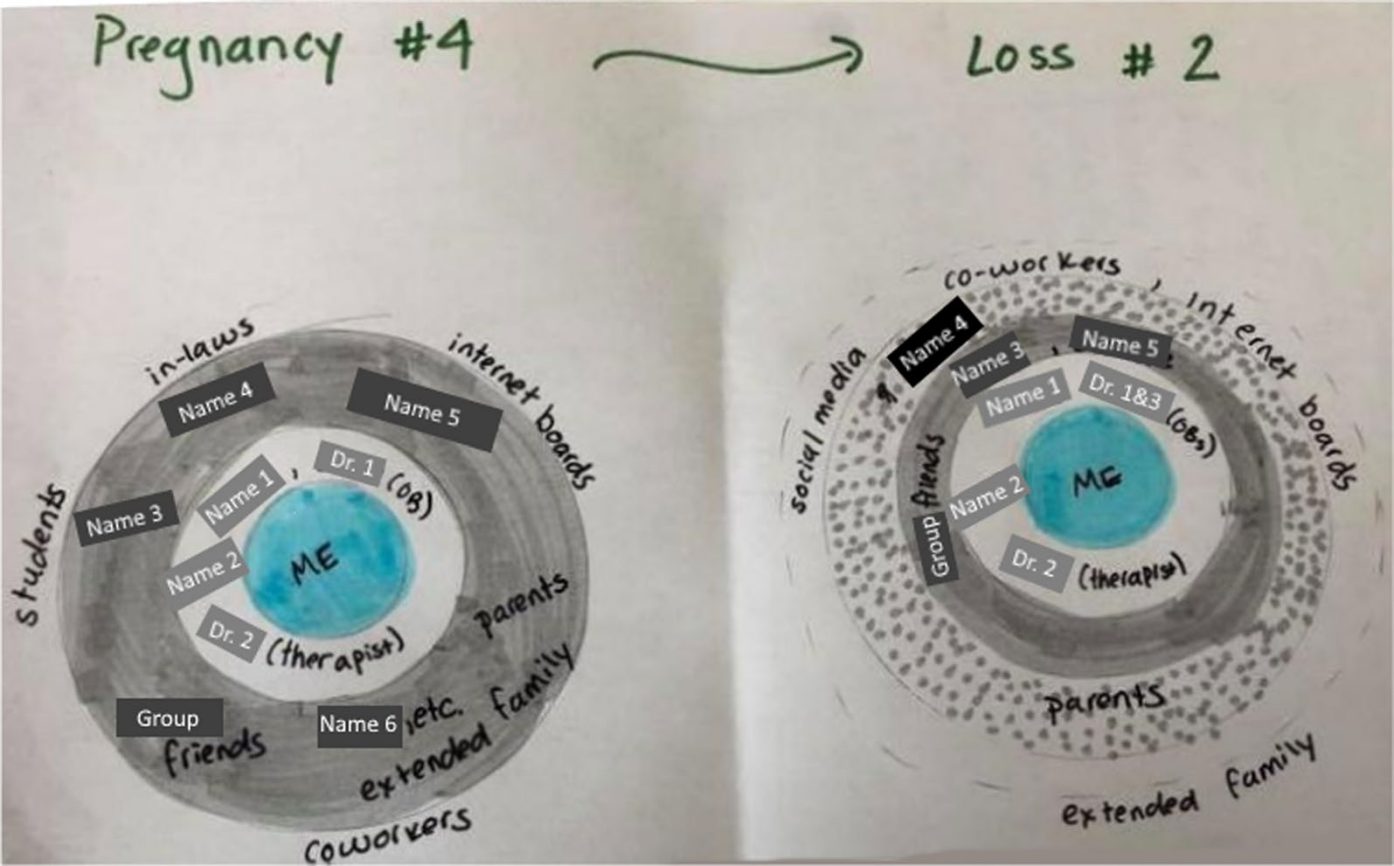
Example of generated circle diagram from activity 7

To identify people in the care network of women who have experienced miscarriage, we triangulated findings from *A7: Circle Diagram* and *A10: Social Support Survey*. In *A7: Circle Diagram*, participants drew a diagram as a series of concentric circles of their social support network when they were pregnant (before miscarriage) and after they miscarried. Participants were asked to place themselves at the centre and people from the formal and informal network who were supportive in circles around the centre, with those who provided more support closer to the centre (c.f. Fig. 1). Additional data was collected in *A10: Social Support Survey*, where participants were asked to identify the people they rely on in specific situations, covering informational, tangible, emotional, esteem and network support situations.

#### Findings

**Fig. 2:**
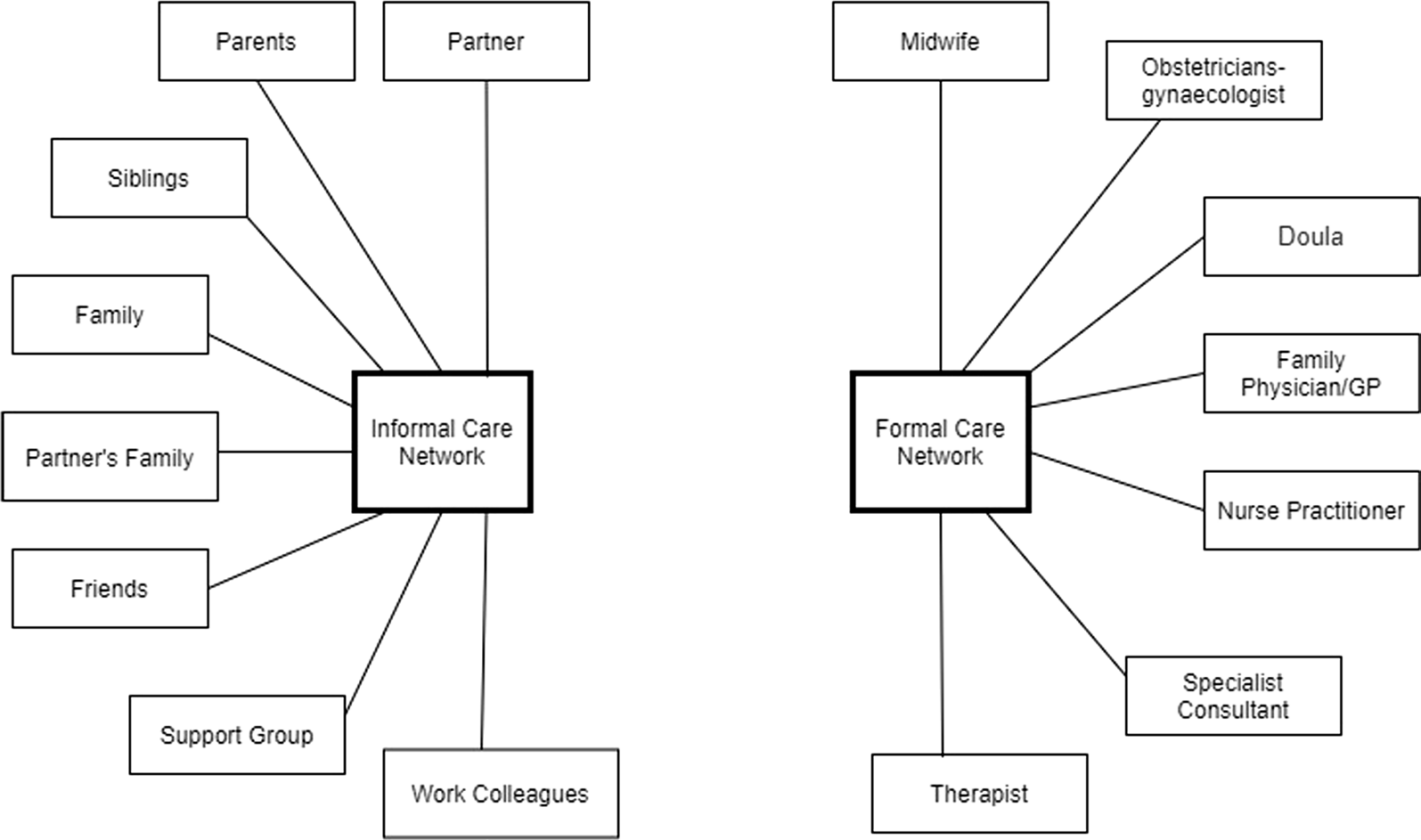
People in participants’ Formal and Informal care networks. The main distinction is between formal and informal support. Both networks are highly multifaceted. There are no generalisable sub-groups; configurations vary by person and by health care system

Participants’ care networks were highly multifaceted. We distinguish two groups, the formal and the informal care network (c.f. Fig. 2). The composition of the formal care network varies between health care systems and women’s individual resources. For example, while all women had a main prenatal care provider, for those in the UK, this was the General Practitioner/midwife, while for those in the US, this tended to be an obstetrician. Some women accessed doulas and therapists, others did not mention them. Likewise, the composition of the informal network depends on women’s overall network and living situation.

**Fig. 3:**
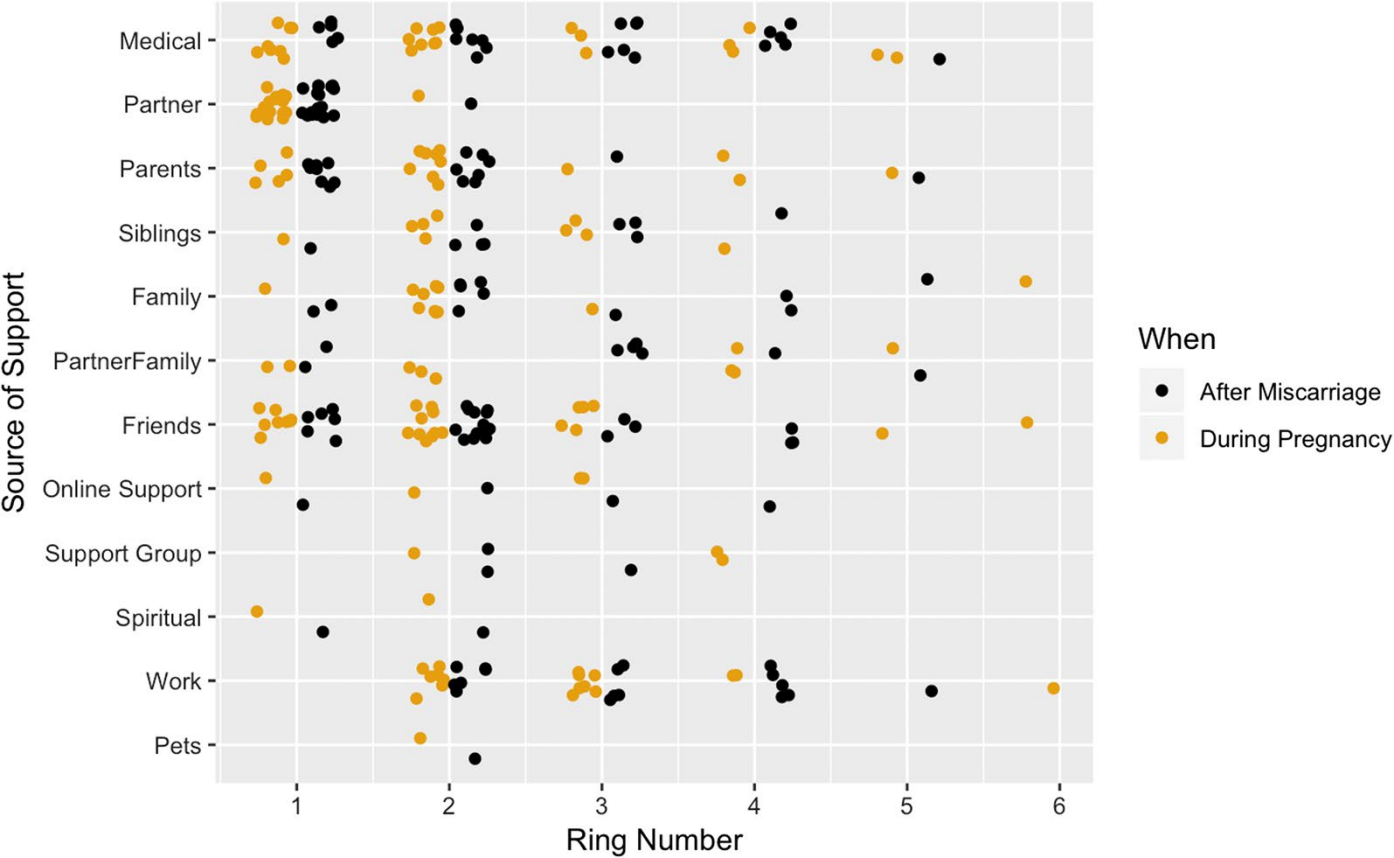
Position in participants’ care network. Lower ring numbers indicate stronger and closer levels of support and involvement. 1 corresponds to the inner ring, 6 is the outermost ring specified by a participants. Positions are shown both during pregnancy and after miscarriage

Figure 3 shows how many participants mentioned a specific type of person in the circle diagram (A7), their ring position during pregnancy, and their ring position after the miscarriage. The lower the ring, the closer the person was to the participant at that time. Both during pregnancy and after miscarriage, participants had an average of 6 support network members in all rings (SD = 3), grouped into an average of 3 distinct rings (SD = 1). These support network members could be individual people or groups of people (c.f. Fig. 3).

The partner, when mentioned, was at the centre of the care network. The relative position of all other groups was far more varied. Four categories were rarely mentioned, but tended to be seen as relatively central (ring positions 1 or 2) when they were: spiritual support, online support groups, in-person support groups, and pets.

When participants were asked about people to whom they could talk about anything related to miscarriage in A10: Social Support, most of the 35 respondents mentioned friends(n = 25, 71%), closely followed by partners (n = 20, 57%). Only 40% (n = 14) listed a person involved in reproductive care services (GP, obstetrician/gynaecologist, midwife). Parents (n = 13, 37%) and other family members (n = 8, 23%) were mentioned less often. Only 5 people (14%) talked about online and in-person support groups.

### RQ2: social support usage and communication with care network

#### Data sources

To better understand how women use social support and communicate with their care network to cope with miscarriage, we used data from *A8: Brief COPE*, *A9: Social Support Communication*, *A10: Social Support Survey*, *A11: Coping Mechanisms* and *A14: Technology Usage*. For *A8: Brief COPE*, participants completed the Brief COPE survey and provided additional free text information about physical and emotional coping strategies. In the Brief COPE, a 28-item survey, people reported the extent to which they used 14 different coping strategies when dealing with a particular situation [84]. These strategies were self-distraction, active coping, denial, substance use, use of emotional support, use of tangible support, behavioral disengagement, venting, positive reframing, planning, humor, acceptance, religion, and self-blame. In *A9: Social Support Communication*, we asked participants to share with the group how they communicated with and sought support from their care network. Additional qualitative information was collected in *A11: Coping Mechanisms*, where participants shared proven strategies that helped them cope with their miscarriage. Finally, as part of the survey *A14: Technology Usage*, we asked participants about trusted sources of information, specifically people (partner, family, health care providers, friends).

When reporting the findings, we begin with an overview of social support usage as coping strategies with miscarriage. We then present in detail our findings on how participants communicate with their care network to receive and seek social support to cope with miscarriage.

#### Coping strategies

The main coping strategy identified by participants in A8 (Brief COPE) was acceptance (c.f. Table 4), followed by use of emotional support, self-distraction, active coping, and use of tangible support. The acceptance was used significantly more frequently than either emotional (Wilcoxon test, Z = 2.5972, $p<0.01$) or tangible support (Wilcoxon test, Z = 3.2963, $p<0.001$). The central role of acceptance as a coping strategy is illustrated by the following quotes from the free text in the supplementary section in A8 (Brief COPE) and A11 (Coping Mechanisms).

**Table 4:** Brief COPE Scale, median and interquartile range (IQR) for No Live Birth (n = 16 responses) and Live Birth (n = 20 responses)

Coping subscale	All participants		No live birth		Live birth	
	Median	IQR	Median	IQR	Median	IQR
Acceptance	3.5	2.5–4.0	3.2	2.5–4.0	3.5	3.0–4.0
**Use of emotional support**	3.0	2.0–3.5	2.7	2.0–3.1	3.0	2.0–3.5
Self distraction	2.5	2.0–3.5	3.0	2.3–3.5	2.5	2.0–3.5
Active coping	2.5	2.0–3.0	2.5	2.0–3.5	2.0	1.5–3.0
Planning	2.5	2.0–3.0	2.5	2.0–3.1	2.5	2.0–3.0
**Use of tangible support**	2.5	2.0–3.0	2.5	2.0–3.1	2.0	2.0–3.0
Venting	2.0	2.0–3.0	2.0	2.0–2.5	2.5	2.0–3.0
Religion	2.0	1.0–3.0	1.5	1.0–2.5	2.0	1.0–3.0
Self blame	2.0	1.5–2.5	2.0	1.0–2.6	2.0	1.5–2.5
Humour	1.5	1.0–2.0	1.7	1.0–2.0	1.5	1.0–2.0
Positive reframing	1.5	1.0–2.0	1.5	1.0–2.0	1.5	1.0–2.0
Disengagement	1.5	1.0–2.0	1.5	1.0–2.0	1.5	1.0–2.0
Denial	1.0	1.0–2.0	1.0	1.0–1.2	1.5	1.0–2.0
Use of alcohol and other substances	1.0	1.0–2.0	1.0	1.0–2.0	1.0	1.0–1.0

Scores range from 1 (not at all) to 4 (a lot)

There will always be a scar for me from these miscarriages, but I have learned to live peacefully with it. [No LB02, Activity A11]

When discussing emotional support, participants mentioned that simply talking to someone who understood what they were going through helped them cope well with both the physical and emotional effect after miscarriage.

Talking to my friends was the thing that saved me. They heard my endless complaints and questions and supported me in my grief. [No LB02, A11]

Tangible support, illustrated in the next quote, often involved the participant’s wider network rallying around them and their partner.

My partner was amazing, and my co-workers took over my work for the week. [LB20, A8]

Although in the qualitative data, women talk more about the need for emotional support than about the need for tangible support, the difference in the Brief COPE scores for the survey between emotional and tangible support was not significant (Wilcoxon test, Z = 0.73784, $p<0.47$). All other coping strategies tended to be used a little bit or not at all. There were no significant differences between the LB and No LB groups regarding acceptance, use of emotional support, and use of tangible support (Wilcoxon test Z = 0.50114, $p<0.62$, emotional support; $\hbox {Z}=-0.99039$, $p<0.32$, tangible support; c.f. Table 4). Moreover, other types of social support, informational, esteem, and network support, were identified from the the qualitative data. Participants highlighted how receiving social support through various means, from health care provider to simply talking and reaching out to someone who understood and validated what they were going through, helped them also cope with miscarriage.

I was already in therapy for other reasons when both of my miscarriages occurred, and meetings with my therapist were very important in helping me cope emotionally. I also reached out to a friend who I knew had multiple miscarriages, and she was very supportive. I also found an online chat group of women who had also experienced loss, which was helpful. [No LB21, A8]

#### Informational support

Most participants wanted more timely information and suggestions on what to do, guidance on what to expect next, stories about other people’s experiences, or at least referral to other resources which would help them navigate their miscarriage journey. A more detailed analysis of these informational support needs is given in [73]. Here, we focus on the role of the informal and formal care network in providing this support.

**Fig. 4:**
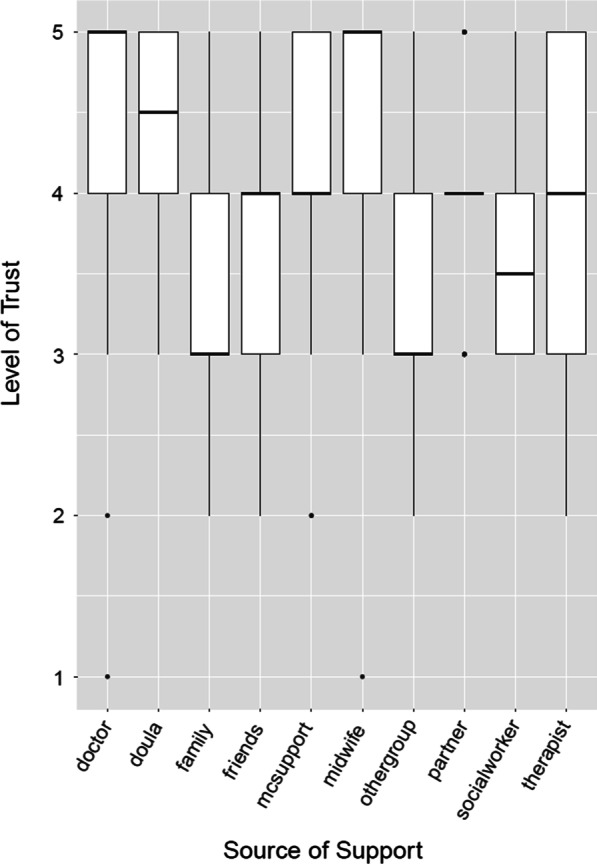
Trust levels for different sources of informational social support. 5 corresponds to “Trust Strongly” and 1 to “Distrust Strongly”. mcsupport = miscarriage support group

*Informal Care Network* The most trusted sources of informational support among the informal care network were miscarriage support groups and partners; partners were also on the innermost ring of the care network in the circle diagram (A7). Family, friends, and other types of support groups were less trusted sources of information (c.f. Fig. 4). Indeed, most participants reported that their informal care network were not sufficiently knowledgeable about miscarriage.

I also wish more medical information was readily available so our support networks can read it and they aren’t relying on hear say and the like. [LB18, A9]

Participants sought to combat this lack of knowledge and the stigma that surrounds miscarriage as it is not a commonly discussed topic, but this could be difficult for some.

My family also isn’t super talkative about loss so there’s that disconnect there on how to handle it. [No LB09, A9]

*Formal care network* The formal care network emerged as a highly trusted source of informational support. Formal health care providers (doctors, midwives) were the most trusted, followed by doulas (c.f. Fig. 4). Providing appropriate informational support by the formal care network can make a substantial difference to a woman’s ability to cope with miscarriage.

Eventually, I found the doctor that made such a difference with my third loss. Through my interactions with her, I was able to overcome my sadness, and feel more positive about my future; mostly because she helped me understand what steps we could take in the future to increase my odds for a successful pregnancy. [No LB23, A8]

However, many participants reported that those trusted sources did not provide them with sufficient information about dealing with miscarriage. The information they hoped to receive involved what to expect next, summaries of other people’s experiences, or at the very least referral to other resources which might help them navigate their miscarriage journey. When the formal care network failed to provide timely informational support, something they are uniquely trusted to provide, it was viewed as a sign they did not care about their patients. Indeed, in A10 (Social Support), less than half of the respondents saw their formal care network as someone to talk to about miscarriage.

The OB [obstetrician] office and its nurses were minimally helpful in term of […] specific suggestions on what to do once the miscarriage starts […]. There wasn’t much guidance in terms of directions and just generally felt like they didn’t care much. Whenever my husband would call the office asking for advice on how to tell how much blood loss is expected, etc, they were not helpful. [LB01, A9]

#### Emotional support

Appropriate emotional support was clearly needed from both the informal and the formal care network of participants. Participants wanted the members of their care networks to have better understanding of their situations, and show empathy so that participants could discuss their feelings openly. Overall, partners played a central role in providing emotional support. Other people who were knowledgeable about participants’ emotional support needs were family, friends, or colleagues who had experienced miscarriage themselves.

*Informal Care Network* Participants often described their partner as very emotionally supportive and having an understanding of the pain of miscarriage.

My husband was super supportive and understanding of allowing me space when I needed it, a shoulder to cry on, and not rushing anything. He was great through all three and was the only one to understand that just because I had gone through it before did not mean I could handle it better or that it was not a big deal. [LB06, A9]

Some participants highlighted the emotional support received from family members, while others discussed friends or co-workers playing a central role in providing empathy and sorrow.

My friends have always been my real family and those who followed also every step of my pregnancies and miscarriages. Always ready to listen, patient, understanding and compassionate. [No LB02, A9]

Participants noted that family members, friends and colleagues who had also experienced miscarriage would be very empathetic and understanding of the participant’s situation. When it was not possible to share thoughts, experiences, or feelings with close friends and family, women with similar experiences filled the gap and were shown to provide valuable emotional support.

I do have a cousin (who is also my coworker) who has had multiple miscarriages so we talked about it a lot together. She was very supportive and is a great resource for me. [No LB20, A9]

On the other hand, some informal care network members expected participants to move past the traumatic and emotional miscarriage event quickly, and did not try to understand what participants were going through, offering up platitudes instead.

Friends and family are so full of platitudes that is is hard. I do not want to hear “it happened for a reason” or “its gods plan” or to be told i should be over ‘it’ by now. [No LB06, A9, original spelling]

Participants often felt unable to discuss their feeling and thoughts openly as they feared a lack of empathy.

I would love to talk about anything miscarriage related, wanting a baby, planning a baby, adoption, everything, with my family but will not. I don’t think the response would be what I need. [No LB09, A10]

*Formal Care Network* The positive experiences that participants reported show that a formal care network that provides appropriate emotional support can make a substantial difference to a participant’s ability to cope with miscarriage in the long term.

My OB [obstetrician] also was helpful and asked apart from medical questions also ones that concerned my psychological state. [No LB02, A9]

Unfortunately, participants often reported a lack of emotional support from the front line formal network they saw at the time of the miscarriage, which potentially made a traumatic experience even worse.

It was incredible difficult times to get through made worse by lack of compassion on my doctors’ and their staff’s part. [LB06, A9]

#### Esteem support

There was substantial evidence that participants did not receive sufficient esteem support during and after their miscarriage experience from either their informal or their formal care network. Participants wanted care network members to be better educated about how to appropriately support someone who has miscarried. The main source of reliable esteem support were others who had experienced miscarriage.

*Informal Care Network* A common characteristic of family members, friends, and colleagues who were described as particularly supportive was that they had also experienced a miscarriage. Women with similar experiences provided much needed esteem support and validated participants’ feelings about their experience.

My colleagues at work were great. Many of them had similar experiences so they knew how to provide support in a helpful way. [LB16, A9]

Sometimes, participants’ experience of loss was not acknowledged as valid by their informal care network, and they received unhelpful comments instead. This lack of esteem support could be quite painful.

Phrases like “when you have kids” are extremely hurtful. We are parents in a different way. [No LB10, A9]

To protect themselves, participants were careful with choosing the people to whom they reached to. It was particularly difficult when no one in their existing informal care network had miscarriage experience.

My mom and mother-in-law have never had a miscarriage and neither have any of my sisters and sister-in-law (only one has had children so far). I am the first one of my close friends to have gotten pregnant so none of then have had losses either. [No LB20, A9]

*Formal Care Network* While there were some instances where participants received adequate informational and emotional support from their formal care network, a failure to validate participants’ feelings towards their loss was viewed as a sign that formal care network members did not care.

We were so hurt. We were shocked by the miscarriage, but more shocked and hurt by the doctor’s response. While it may have been his umpteenth miscarriage, it was our first. [LB23, A9]

#### Network support

Participants wanted timely and easy access to support groups and other people with similar experiences, which provided a sense of belonging. Unfortunately, such groups were often difficult to find.

Relatively few participants mentioned in-person and online support groups in the Circle Diagram (A7), and tended to place them in Rings 2 and 3. However, whenever support groups were mentioned in any of the free text responses, participants talked extensively about the positive role of these groups.

*Informal Care Network* Participants noted that the shared loss created a strong network connection to those who had also experienced miscarriage. Online and in-person miscarriage support groups created a safe space where participants felt understood, and that engendered a sense of belonging and community that participants were sometimes unable to obtain elsewhere.

Some extended family and some of my friends’ moms have had miscarriages and have reached out to me as well. [No LB20, A9]

Some participants proactively built their own network support, which complemented support groups organised by charities.

I also reached out to a friend who I knew had had multiple miscarriages, and she was very supportive. I also found an online chat group of women who had also experienced loss, which was helpful. Finally, after my second miscarriage, I learned about some local in-person support resources and has been one of the most important support systems for helping me cope. [No LB21, A8]

While appropriate network support was very important when helping participants cope after miscarriage, it could take time to find it. Putting participants in touch with support groups or with other people with similar experience at the right time was key for the healing process.

Finally, after my second miscarriage, I learned about some local in-person support resources (burial ceremony, monthly support meetings), which I am still attending through my current pregnancy, and has been one of the most important support systems for helping me cope. [No LB21, A8]

*Formal Care Network* Participants wanted their formal care network to signpost them to reliable sources of network support.

It would have been nice if the [Health Care Provider] had a network to reach out and tell me about emotional support groups. [No LB08, A9]

#### Tangible support

**Fig. 5:**
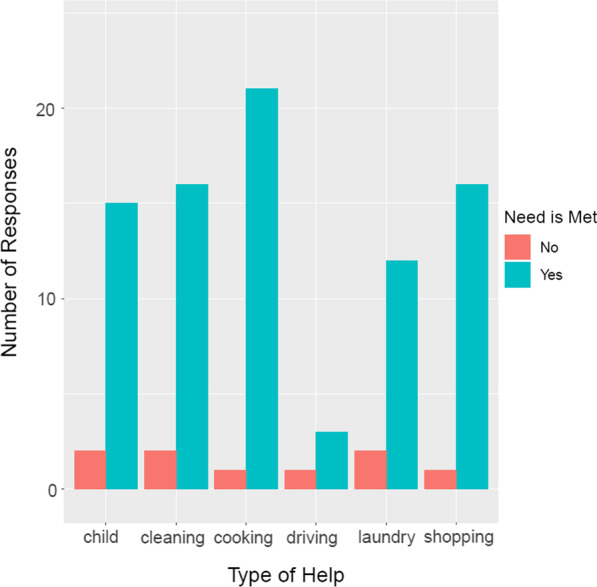
Need for tangible support. Number of responses that indicated the need for help with a chore. If the need is met, there is a person in the woman’s care network who can help with the chore; if the need is not met, there is not

We explored the availability of tangible support in detail in the Social Support survey (A10). Participants were asked about seven types of tangible support: cooking, laundry, cleaning, child care, driving, shopping, and pet care. None of the participants who took part in this activity were interested in pet care, but all stated that they would like help with at least one of the other activities. Participants named around 3 activities that they wanted support with (median: 3, inter-quartile range: 3–4). Figure 5 shows the relative popularity of each type of support. Cooking/meals was the most requested type of tangible support, followed by help with shopping, cleaning, and child care. Tangible support was only provided by the informal care network. Participants did not report any schemes for providing tangible support post-miscarriage that had been set up through their formal care network.

Participants were satisfied with the tangible support received. Most participants who stated a need for tangible support had someone in their informal care network of friends and family who could meet it (c.f. Fig. 5).

My co-workers took over my work for the week. It was hard going back to work the following week, emotionally.[LB20, A8]

While some had a wide network of helpers, others only had their partners, and a few participants wanted their informal support network to be a little more proactive in reaching out to them.

#### Summary

When participants did receive appropriate social support, they benefited substantially. Unfortunately, sometimes, it was difficult to find the sources of social support, in particular esteem and network support, and sometimes, they were only found during a later miscarriage experience. While the informal care network was mostly responsible in providing appropriate tangible support, there was substantial evidence that the provision of informational, emotional, esteem, and network support during and after their miscarriage experiences was patchy. Often, participants experienced a disconcerting mix of reliable social support from some trusted sources such as partners, and a lack of social support from others, especially the formal care network and members of the informal care network without miscarriage experience.

### RQ3: technology usage to facilitate coping and seek social support

#### Data sources

To explore how eHealth might be used to improve social support communication, we used data from *A9: Social Support Communication* and data from the survey *A14: Technology Usage*. We asked participants which technology they currently use to communicate with others and receive different types of social support.

#### Findings

**Fig. 6:**
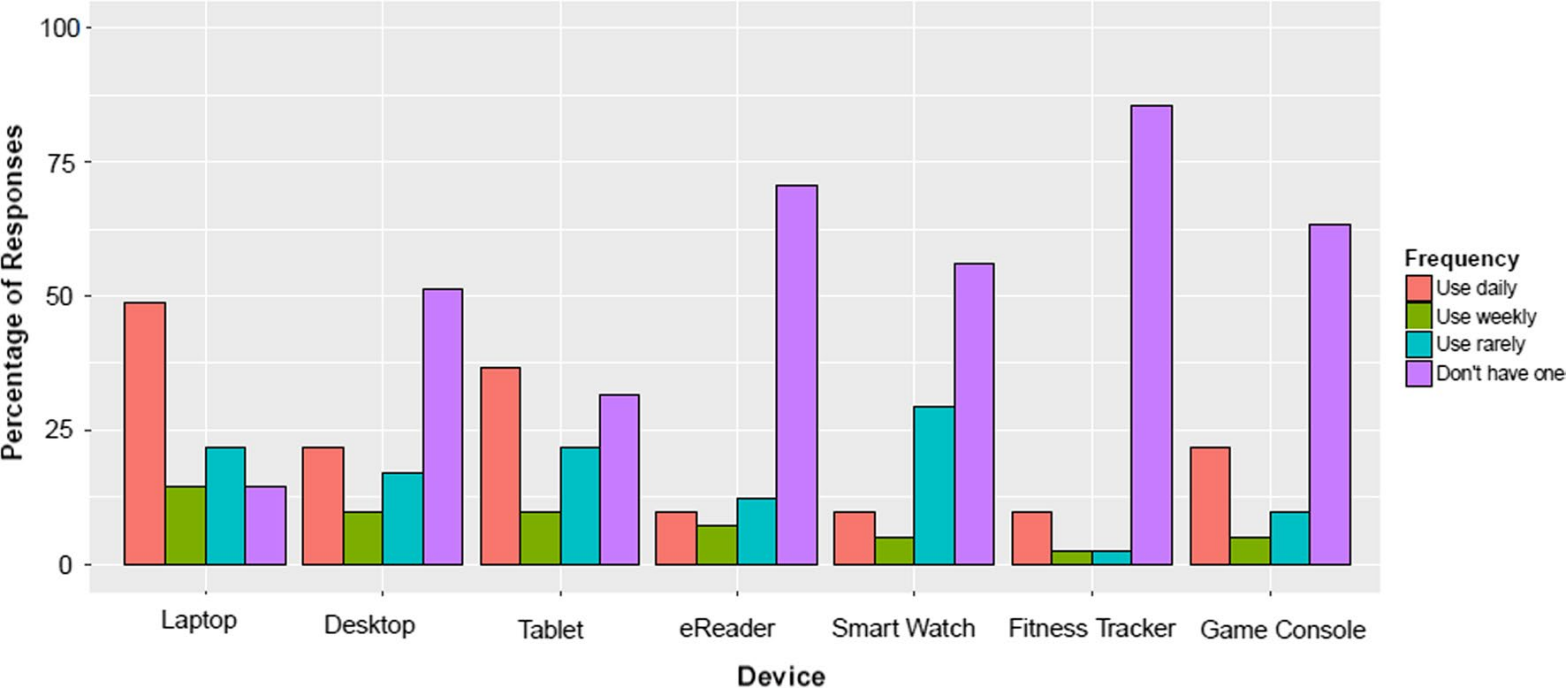
Use of devices. Number of participants in demographics survey who indicated that they had a laptop, desktop, tablet, eReader, smart watch, fitness tracker, or games console

**Fig. 7:**
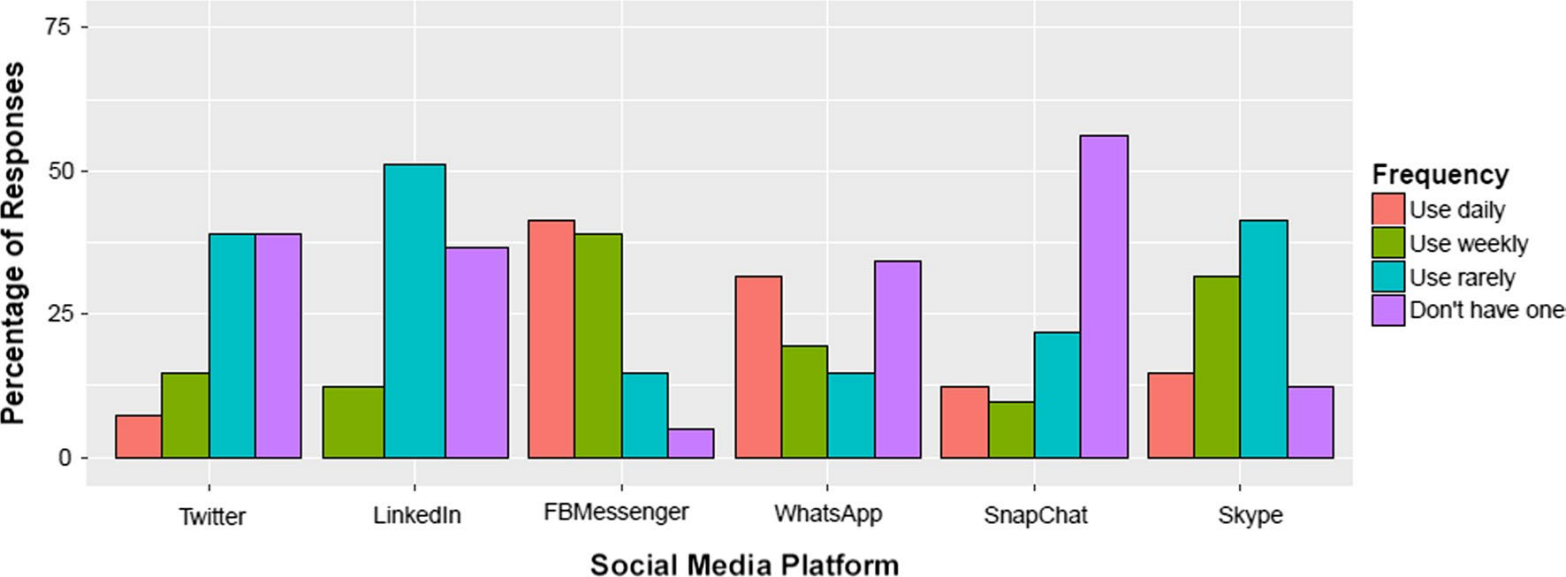
Use of social media accounts. Number of participants in demographics survey who indicated that they had an account on Twitter, LinkedIn, Facebook Messenger, WhatsApp, Snapchat, or Skype

Participants’ device use and social networking footprints, as reported in the demographic survey, were almost as diverse as their care networks. Most participants also had a laptop, and two thirds owned a tablet, as shown in Fig. 6. All other devices were less common, and used far less often. With respect to social networking account use (c.f. Fig. 7), most participants have a Facebook Messenger account. Skype was the second most common additional account, followed by WhatsApp. Moreover, all participants had a smartphone, and almost all used Facebook daily. However, this was the only technology use pattern they had in common.

If support was needed that was not provided by their in-person care network, participants turned to online resources and communities. The results of A14 (Technology Usage), as summarised in [79], show that participants used blogs mainly as a source of informational support around miscarriage, health care, and recovery. Facebook was a source of emotional support, while online forums were important sources of informational, emotional, and tangible support.

I sought the support of my online ’community’ friends, many of whom had gone through similar, and it was a huge help to me. [LB12, A8]

Overall, online support groups were considered to be knowledgeable about participants’ needs and provided comfort and support. Online networks played an essential role during women’s grieving process, allowed women to feel less alone, and gain much needed comfort. Participants proactively sought informational and emotional support through online resources, forums, and communities.

The people in my online care network are very knowledgeable about my needs, as there are so many different friends there who have gone through all sorts. [LB12, A9]

Our findings show that participants used technology in many different ways to remove the barriers of communication with their care network. Some participant employed online platforms to eliminate the stigma surrounding miscarriage and notify their care network about their miscarriage, while others used online spaces to educate their care network and raise awareness of the pain of miscarriage.

I posted information about how basically losing a pregnancy is hard and just as hard as losing a friend or family member and to think about the phrasing used. [No LB06, A9]

However, it seems that social media and other internet support boards can do harm as well as good, in particular immediately after loss, and when participants made decisions that were not supported by the groups to which they belonged.

I never talked on social media or any support groups at the time and generally wanted to get over it just by being with my loved ones who knew every single bit of the story. [No LB02, A9]

Although participants sought different social support sources, they typically focused on a few online sources, especially those that confirmed they were not alone. While online support is an important and promising option, it clearly needs to be tailored to the social media platforms and online resources with which they feel comfortable. There was no single forum, app, or platform that was unequivocally endorsed by all women who used it—not even the platform they all had in common, Facebook.

## Discussion

Our findings highlight the complexity of the formal and informal care networks that are involved in providing social support to women who have miscarried. The ARC method allowed us to explore this complexity in depth through a sequence of guided activities that brought together people with different backgrounds and personal histories. The online asynchronous format made it possible for participants to engage with activities in their own time, without having to tell anyone that they were engaging in reflection and research on miscarriage.

While the support functions of each part of the formal and informal care network overlap, our results show clearly that the formal care network dominates when it comes to informational support, and the informal care network dominates when it comes to tangible support. When it comes to emotional, esteem, and network support, however, participants describe receiving better support from similar others [14, 57] (i.e., people who also had experienced miscarriage who were more familiar with their experiences and better able to empathise). Despite the differences in participants’ individual social support needs, women who have experienced miscarriage have many challenges in common [14]. Participants identified that providing one type of support was not sufficient to make participants feel truly supported, and all types of support were valued.

The potential role of technology and online spaces in facilitating access to social support is complex. Participants in this study provided many examples of how they communicate with their care network through various means. Some of these examples were person to person (e.g., talking to the therapist, meeting with a friend), others were shared with more than one person (e.g., posting on Facebook). Some are synchronous (e.g., a phone call to a midwife) and some asynchronous (e.g., text family). While Internet and social media were often useful sources of such connections and information, as observed in the literature [29, 57, 65], they were not the only ones. Participants still valued in-person support. Indeed, tangible support, which is by definition delivered in person, was both valued and also provided reliably.

Since participants have many people in the care network who perform different roles, and since participants actively used and coordinated different channels (people, social media, other internet resources) to obtain the support they needed, we require a model that allows us to accommodate this degree of complexity. The Circle of Care framework, introduced in “Shifting towards a circle of care model” section, fits the requirement. The CCM approach has shown success in end-of-life patient communities (e.g., [70]), but has not yet been explored as a way of facilitating social support between women who have miscarried and their care networks. We see this approach as having potential benefits for miscarriage communities, for whom social support is limited and difficult to coordinate.

### Implications for service design: the miscarriage circle of care

**Fig. 8:**
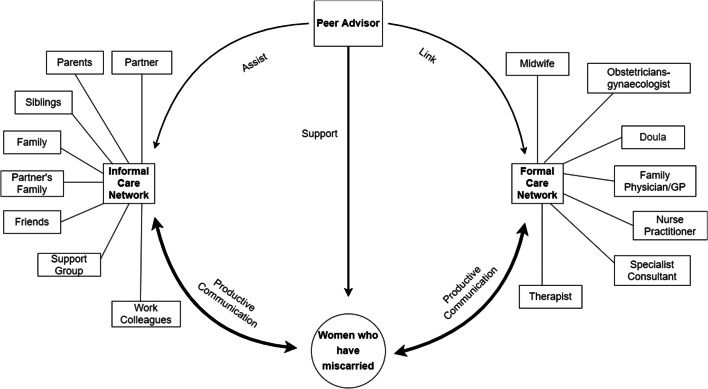
Miscarriage circle of care. Peer Advisors integrated into care networks to enabling better social support communication among the miscarriage circle of care

A Circle of Care Model (CCM) allows members of care networks to act in a variety of support roles, such as providing information, validating the feelings of those who have miscarried, or helping with chores. It also explicitly incorporates resources that can be drawn on to do so, such as information about recovery and miscarriage, or links to support groups. When outlining a CCM in the context of specific health care service provision, it is also possible to address issues, such as the timely provision of social support. In a previous analysis of women’s information needs, drawing on the same data set as this study [73], we identified that timely provision of information requires close coordination with the individual person’s miscarriage timeline, and the service providers that are available at each stage. Figure 8 shows how our findings can be mapped onto an initial Miscarriage Circle of Care Model (MCCM). The MCCM offers a logical structure of connected concepts that help display how the social support and the support resources (i.e., care networks) might be connected and relate to one another. It also gives us an opportunity to specify and define the communication framework necessary to address the social support needs of women who have miscarried.

The communication framework in the MCCM draws on formal and informal care networks and “Peer Advisors” to add further depth and breadth to our understanding of social support communication for women who have experienced the miscarriage circle of care. Productive communication between the women who have miscarried and the networks who can provide the support they need is the central challenge to be addressed. With productive communication, members of the informal care network learn how to provide effective emotional and esteem support, and members of the formal care network can leverage the pivotal role they play in women’s miscarriage experiences to provide effective informational, emotional, and esteem support. With a clear focus on productive communication, we can proceed to understand the barriers that prevent women who have miscarried from accessing effective social support, and put in place measures that overcome those barriers. Our findings suggest that measures should in particular target reducing stigma and ensuring that women do not feel alone.

We view peer advisors as the key to facilitating such productive exchanges. They *support* the woman who has miscarried, they can *link* with relevant members of the formal care network, and they can *assist* members of the informal care network in facilitating the support that is actually needed. As previous work on peer support for miscarriage by telephone has shown, peer advisors should in turn be supported in their role [85], which can be through a dedicated peer advisor network or links to healthcare providers.

The MCCM currently does not include links within the formal care network and links within the informal care network. For the formal care network, the network-internal webs of connection depend on the health care system where the woman lives, the existing communication channels between providers, and whether these are in person, by letter, by phone, by email, or via record linkage. The informal care network is even more complex. Some parts may be missing (e.g., work colleagues for women who are primarily homemakers), and some parts can be multifaceted in themselves (e.g., different groups of friends).

While communication between members of the formal care network is likely to be highly regulated, and require technology that can adhere to those regulations, communication between members of the informal care network can flow across multiple social media platforms, in addition to phone calls, texts, and in-person meetings. Tighter integration of these networks is beneficial, since this relieves the load on individual support providers [68]. Communication within the care networks is crucial, as gaps in communication can lead to unmet needs and, in the worst case, adverse events [72].

CCM models of the formal care networks, which have been created for areas such as end of life care [71], might also be useful in embedding appropriate social support practices within the formal care network. Even though researchers and practitioners have repeatedly called for, and developed approaches to deliver, improved social support in miscarriage care (e.g. [86], our findings show that this knowledge does not translate into practice. We suspect that part of the reason is structural. Providing miscarriage care is demanding, and members of the formal care network need to be properly supported in the work they do [87, 88].

### Limitations

Several issues affect the generalisability of our findings. The number of participants per group and per health care system, as indicated in Table 2, is too small for reliably detecting medium-sized effects. The participants in our study were predominantly white, well educated, and established Facebook users, which might be reflected in their use of Facebook as a resource to provide support. Despite our efforts, it was difficult to reach people from lower socioeconomic strata, and people from non-white populations. As Fig. 7 suggests, online social media use is highly fragmented, which means that there is no single social media channel that would allow us to reach everyone who is online. Therefore, a different platform, such as WhatsApp, might have given us access to a different population.

In our study, we split participants into two groups depending on whether they had experienced a live birth after their last miscarriage or not in an effort to ensure somewhat similar reproductive health histories, however, this may have been too little differentiation. The No LB group, which had lower participation rates, included both women who already had children and women who had decided not to try to conceive again after their last loss. Some participants had a history of recurrent miscarriages, while others had undergone fertility treatment. Our study also focused on the person experiencing miscarriage, not on the experience of the people in their formal and informal care networks (e.g. partner), who often also need support [89–91].

Finally, the stories the participants shared took place over several decades, with some being very recent. We acknowledge that health services are fully aware of the need for better informational and emotional support, and are taking steps to provide this [89, 92].

## Conclusion

This study provided insight into the social support needs of women who have miscarried, and their perspective on communicating with their formal and informal care network about needs. Just as comprehensive social support in pregnancy has been shown to decisively improve perinatal mental health [93], well-designed social support for those who have miscarried has the potential to substantially improve outcomes [86], but there are still substantial gaps in the literature, especially when it comes to Randomised Controlled Trials that inform care guidelines [94], and in implementation.

In order to improve miscarriage care, we proposed adopting the Circle of Care Model approach and suggested an initial communication model between formal and informal care networks (Miscarriage Circle of Care Model), where peer advisors play a pivotal role in facilitating social support, and in enabling people to carry out the roles they have in the circle of care. While there is a clear need for technology that links women to people in the Circle of Care, and helps them meet their varied social support needs, the requirements for this technology are complex, and range from adapting Electronic Health Record systems to providing mHealth solutions that can improve the communication between women who have miscarried and their care network [95, 96].

In future work, we will further refine and expand the MCCM to design mHealth solutions that allow seamless access to social support, and to the people who can provide this support, as and when needed. Such solutions need to be co-designed with women who have miscarried, and their formal and informal care networks, as identified in the MCCM.

## Data Availability

The datasets generated and/or analysed during the current study are not publicly available due to the sensitive nature of the data, but fully anonymised versions are available from Dr Maria Wolters (maria.wolters@ed.ac.uk) or Prof Katie Siek (ksiek@indiana.edu) on reasonable request.

## Abbreviations

A: Activity during the Asynchronous Remote Community online Focus Groups
ARC: Asynchronous Remote Community
CCM: Circle of Care Model
LB: Participant group with Live Birth after most recent miscarriage
MCCM: Miscarriage Circle of Care Model
NLRD: Network-Level Reciprocal Disclosure
NoLB: No Live Birth after most recent miscarriage
RQ: Research Question
UK: United Kingdom
US: United States of America

## Footnotes

1 https://www.r-project.org.

2 https://www.dedoose.com.
